# Tissue-specific expression of TRP channel genes in the mouse and its variation in three different mouse strains

**DOI:** 10.1186/1471-2164-7-159

**Published:** 2006-06-20

**Authors:** Christiane Kunert-Keil, Frederike Bisping, Jana Krüger, Heinrich Brinkmeier

**Affiliations:** 1 Ernst Moritz Arndt University of Greifswald, Institute of Pathophysiology, Greifswalder Str. 11C, D-17495 Karlsburg, Germany

## Abstract

**Background:**

The purpose of this work was to study the gene expression of transient receptor potential (TRP) channels in the mouse. The application of a standardized and quantitative technique, TaqMan RT-PCR, should give information about the pattern and relative importance of TRP channels for murine tissues and cell types. To verify data sets with an independent method, we studied the occurrence of some of the transcripts by *in situ* hybridization.

**Results:**

We have characterized the mRNA expression of 22 TRP channels in the mouse with a focus on nerve and muscle tissues. This is the first study to describe the expression profiles of all channel isoforms of the four related Group 1 subfamilies (TRPC, TRPV, TRPM and TRPA) with a standardized and quantitative technique. Comparisons of transcript abundance showed a consistent dominance of TRPM7 and TRPC3 in most tissues. We further observed characteristic patterns and differences in gene expression of individual channels ranging over three orders of magnitude. The overall level of TRP channel mRNAs was highest in brain areas followed by kidney, lung, reproductive organs and muscle. In brain TRPM3 and TRPM7 dominated and 19 other isoforms were detected. In lung and kidney TRPV4, TRPV5 and TRPM7 were found in highest levels. TRPM7, TRPC3, TRPC6 and TRPM3 mRNAs were characteristically present in all tested muscle tissues. Most data obtained with the C57Bl/10 mouse strain were confirmed with Balb/c and NOD mice. However, TRPC3, C6, TRPM7, M3, TRPV2 and V4 expression showed marked differences in the three tested mouse strains. *In situ *hybridization revealed co-expression of transcripts on the cellular level and widely confirmed the data obtained with RT-PCR.

**Conclusion:**

Transcripts coding for members of the TRPC, TRPV, TRPM and TRPA subfamilies of TRP cation channels are present in a broad spectrum of murine tissues. Several channel isoforms often coexist in a specific tissue or cell type. TRP channel expression does not show typical tissue specific dominance of individual members as is known from other ion channel families. Mouse strain specific variations of TRP channel expression indicate that genetic background or physiological requirements considerably influence expression levels.

## Background

The mammalian homologues of the Drosophila transient receptor potential (TRP) channel are plasma membrane cation channels sharing significant sequence homology and a common structure. In mice 28 genes have been described coding for TRP proteins [1]. The superfamily of TRP channels has been divided into six subfamilies in mammals, TRPC, TRPV, TRPM, TRPA, TRPP and TRPML according to structural similarities and modes of activation [1,2]. The members of subfamilies TRPC, TRPV, TRPM and TRPA comprise the Group 1 TRPs, since TRPP and TRPML members are distantly related and classified as the Group 2 TRPs [1]. TRP genes are almost ubiquitously expressed and occur in splice variants [3,4]. The channel proteins interact with a variety of other proteins to form macromolecular complexes [5]. The selectivity for specific cations is diverse among TRP channels. Some of them are selective for Ca^2+^, some for Na^+ ^or Mg^2+^, while others seem to be non-selective cation channels. The known functions of TRP channels are extremely diverse as well. Some of them mediate sensory transduction of cold, heat, mechanical, osmotic or noxious stimuli [6]. Others are involved in the regulation of neuronal function, vascular tone [7], epithelial calcium absorption and the regulation of cellular calcium and magnesium homeostasis [2,8]. The variety of isoforms and splice variants and the fact that one cell type often expresses several types of TRP proteins makes it difficult to elucidate the precise function of TRP channels in individual cells types and tissues. In some cases, one channel isoform is dominant in a specified cell type, i.e. it can be detected in relatively high levels by RT-PCR, *in situ *hybridization and immunochemical methods. In these rare cases TRP channel expression can be correlated with physiological function such as inward currents of cations and the modulation of cellular excitability or calcium homeostasis.

As the majority of TRP channels allows an influx of calcium into the cell and since calcium is the most common cellular second messenger, TRP channel function may have important consequences for cell growth and division, differentiation and also for cell damage and cell death [9,10].

To study the roles of TRP channels in cells and tissues, it is important to know where they are expressed and at what level they are expressed. In order to obtain valid data about the tissue specific expression it can be important and helpful to test gene expression with several independent techniques. Unfortunately, the current expression studies in mammals are heterogeneous with regard to species, applied methods, number of included tissues and cell types and number of included TRP channels. Some authors show the expression of several TRPC isoforms in a variety of rat tissues and cell lines [11], some focus on specific tissues as vessels or vascular smooth muscle [7] or human tissue samples of the central nervous system [12]. Most studies cover only one subfamily of TRP channels, e.g. TRPC [11-13] or TRPV [14], although in many cell types channels of several families are expressed and certain members of different families may have overlapping physiological functions. At present it is not clear whether TRP channels show a characteristic tissue-specific expression that is constant in different mammalian species or mouse strains.

The current study was designed to prepare a basis for investigating the relevance of TRP channels in mammalian tissues in health and disease. We determined the expression of 22 TRP channel genes in the mouse, one of the major model organisms of man. Besides the method of RT-PCR, we used the quantitative method of real-time RT-PCR to investigate the relative expression levels of TRP channel genes in a broad spectrum of tissues in three mouse strains. This should give information about the relative significance of distinct TRP isoforms in a specific tissue and address the question whether redundancy is a characteristic feature of TRP channels. Before doing functional studies it is important to know whether there is a dominating channel expressed in a certain tissue or cell type. To verify the data obtained with real-time RT-PCR using an independent method, we applied the technique of *in situ *hybridization. Although we focus on nerve and muscle tissue, an almost complete set of data for a wide spectrum of mouse tissues is presented.

## Results

### Detection and quantification of TRP channel transcripts in mouse tissues

Using standard RT-PCR as well as real-time RT-PCR we detected signals for all above mentioned TRP channel transcripts in many murine tissues. We observed characteristic expression patterns and differences in gene expression of individual TRP channels ranging over three orders of magnitude.

**Figure 1:**
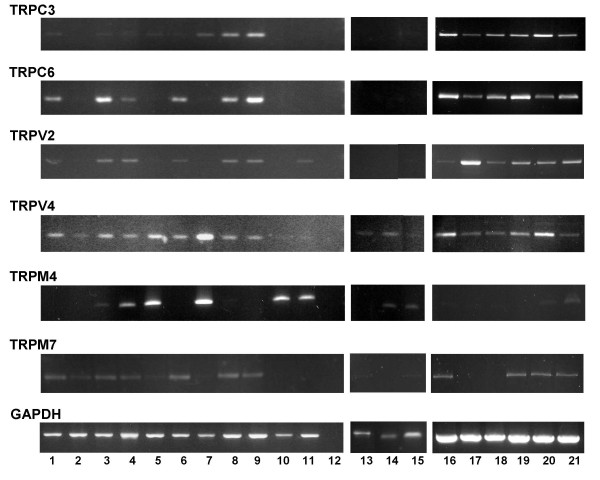
**Expression of TRP channel mRNAs in murine tissues using standard RT-PCR**. Reverse transcribed RNA (8 ng) from tissues of adult (100 d) C57Bl/10SC mice was added to the reaction mixtures and PCR products amplified in 40 cycles. The products were separated on agarose gels and stained with ethidium bromide. Water served as negative control. 1) testis; 2) liver; 3) lung; 4) spleen; 5) skeletal muscle; 6) epididymis; 7) kidney; 8) ovary; 9) seminal vesicle; 10) uterus; 11) C2C12 cells; 12) H_2_O; 13) aorta; 14) intestine; 15) heart; 16) hippocampus; 17) brain stem 18) forebrain; 19) cerebrum 20) cerebellum; 21) truncus encephali.

Standard RT-PCR of TRP channel transcripts from peripheral tissues and brain regions from Balb/c mice as well as from the mouse skeletal muscle cell line C2C12 yielded PCR products of expected size. All products were sequenced in an exemplary manner and revealed the expected DNA sequence. Figure 1 shows the results for some frequently observed TRP channels, TRPC3, TRPC6, TRPV2, TRPV4, TRPM4, TRPM7 and GAPDH, respectively. All tested tissues were positive for TRPV4 mRNA coding for a cation channel sensitive to extracellular osmolarity. The strongest response was observed with kidney (Fig. 1, line 4, column 7). The occurrence of TRPM4 mRNA showed the largest variation among the tested tissues and, in contrast to all other transcripts a low expression in brain regions (Fig. 1, columns 16–21). The tested brain regions were consistently positive for TRPC3 and 6 as well as for TRPV2 and 4 mRNAs. Brain regions showed the overall strongest expression of TRP channel transcripts and at the same time the highest diversity of TRP channel gene expression. Muscle tissues, aorta (col 13), heart (col 15) uterus (col 10) and skeletal muscle (col 5) were positive for few TRP mRNAs, in particular TRPC3 and TRPV4, but the signals were relatively weak.

In order to quantify tissue specific TRP channel expression more precisely we applied TaqMan RT-PCR on tissue samples from adult C57Bl/10 mice. For the murine transcripts of TRPC3 and 4, TRPV1 and 5 as well as for TRPM2-4 and TRPM6 specific TaqMan primers and probe sets were developed. They were used to prepare DNA standards and to perform real-time RT-PCR. For all other transcripts coding for TRP channels commercially available primers and probe sets were used. The primer pairs were chosen in order to obtain products that cross intron-exon borders to exclude results from a contamination with genomic DNA. Parallel to the analyses of TRP channel transcripts, the copy number of eukaroytic 18S rRNA was determined for each individual sample. Only minor differences in the expression of this housekeeping gene were observed between the different tissues (data not shown), suggesting that 18S rRNA can be used for normalization.

**Figure 2:**
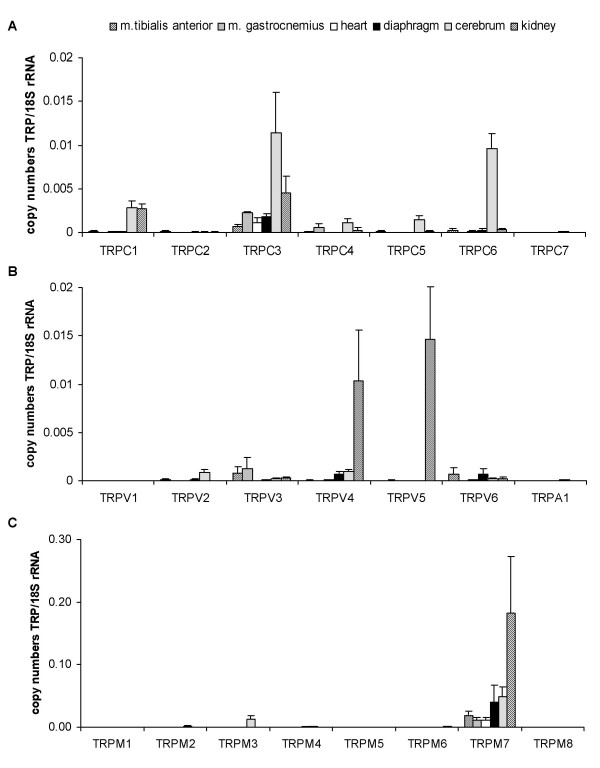
**Quantification of TRP ion channel mRNA levels in murine tissues using Real-time RT-PCR**. Expression of TRPC1-7 (A), TRPV1-6 and TRPA1 (B) as well as TRPM1-8 (C) mRNA was analyzed in murine tissues using Real-time RT-PCR. Samples were from adult (100 d) C57Bl/10SC mice. The mRNA levels of TRP ion channels are given in relation to 18S rRNA. Means ± S.E.M. are given in all cases for n = 3–10 samples.

**Figure 3:**
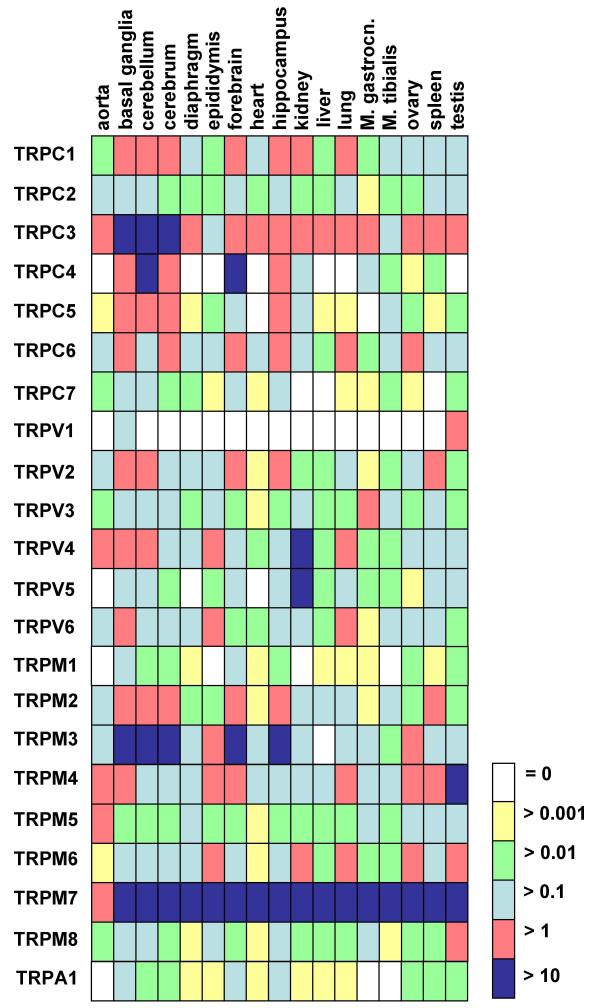
**Summary of tissue specific TRP channel gene expression**. Expression of TRPC1-7, TRPM1-8, TRPV1-6 and TRPA1 mRNAs in murine tissues was determined by real-time RT-PCR. Levels of mRNAs are given in relation to that of 18S rRNA; all data were multiplied by 1000. Means were calculated from n = 3–10 samples and classified into 5 categories. If the threshold level of fluorescence according to the TaqMan RT-PCR technique was not reached after 40 cycles of amplification, a resulting expression level of zero (0) was assigned to the corresponding channel transcript and tissue. For better illustration categories of gene expression levels are represented by different colors ranging from zero (white) to values higher than 10 (dark blue).

The strongest expression of TRPC channels was found in brain areas. In cerebrum TRPC3 and TRPC6 dominated followed by TRPC1, 4 and 5. TRPC2 and 7 only occurred in low concentrations reaching 2% of that of TRPC1 (Fig. 2A and 3). TRPC1 mRNA showed a rather constant level in all tested brain areas while other isoforms varied considerably. TRPC6 mRNA was very prominent in cerebrum and basal ganglia but much less expressed in cerebellum. In the hippocampus TRPC3 was the most abundant isoform among the TRPCs. TRPC channel transcripts were also prevalent in peripheral tissues. In the muscle tissues, skeletal muscle, aorta and heart, the TRPC3 transcript dominated over all other members of the TRPC subfamily (Fig. 2A). The next most frequent isoforms were TRPC1 and TRPC6 reaching about 10% of the level of TRPC3 in heart and diaphragm (Fig. 3).

Members of the TRPV subfamily of cation channels are known for their functions in sensation of heat, cold and other stimuli (TRPV1-4) and for Ca^2+ ^transport through the epithelia (TRPV5-6). In this study TRPV1 mRNA was hardly detectable in any of the tested tissues and only found at significant levels in basal ganglia and testis. TRPV2 was the dominating isoform in all tested brain areas. Among the TRPVs only TRPV4 reached similar levels. However, compared to the TRPC transcripts, TRPV2 and 4 mRNAs were expressed at much lower levels reaching only 3–10% of those of TRPC3 and TRPC4 (Fig. 2B and 3). As expected, strong signals were found for TRPV5 in kidney and TRPV6 in lung. In addition TRPV4 transcripts were found at high levels in these two organs. Altogether in lung and kidney transcripts coding for TRPV channels dominated over those of TRPCs. In muscle tissues transcripts of TRPV3, 4 and 6 were regularly detected. While TRPV3 showed quite a strong expression in tibial and gastrocnemius muscle, TRPV4 and TRPV6 were the most abundant isoforms in diaphragm and aorta. In heart muscle the expression the TRPVs was very low (Fig. 2B and 3).

Channels of the TRPM subfamily serve different functions as they are involved in cold sensation (TRPM8), Mg^2+ ^transport (TRPM6 and 7) and can have an enzymatic function in addition to their channel activity (TRPM2, 6, 7). In the mouse, the TRPM7 gene was consistently expressed in all tested organs and tissues and it was at the same time it was the dominating isoform except for brain areas. In brain TRPM3 mRNA was found at highest levels and TRPM2 transcripts were also found at considerable levels reaching about 10% of those of TRPM3 and 7 (Fig. 2C and 3). In muscle tissues TRPM7 dominated, followed by TRPM3 and 4. The highest expression and diversity among TRPMs was observed in the aorta, while heart muscle showed the overall lowest expression (Fig. 2C and 3).

The TRPA1 transcript coding for a channel possibly involved in cold and chemosensation was found in low levels in various brain regions as well as in reproductive organs and spleen (Fig. 2B and 3).

Taken together, brain areas showed a high diversity and strong expression of TRP channels. Most prominent were TRPM7 and M3 and TRPC3 and C4, but many other isoforms are potentially relevant as their transcripts occurred at significant levels. Kidney revealed a more restricted and characteristic pattern of TRP channels dominated by TRPM7, TRPV4 and V5, followed by TRPC3 and C1. Muscle tissues showed much lower expression, at least one order of magnitude lower than brain and kidney. There seem to be differences in TRP channel expression between smooth muscle (aorta), skeletal muscles and heart but it is difficult to recognize a typical tissue-specific pattern that is characteristic and differentiates the three muscle tissues.

### Cellular localization of TRPC3, TRPC5 and TRPC6 mRNAs

**Figure 4:**
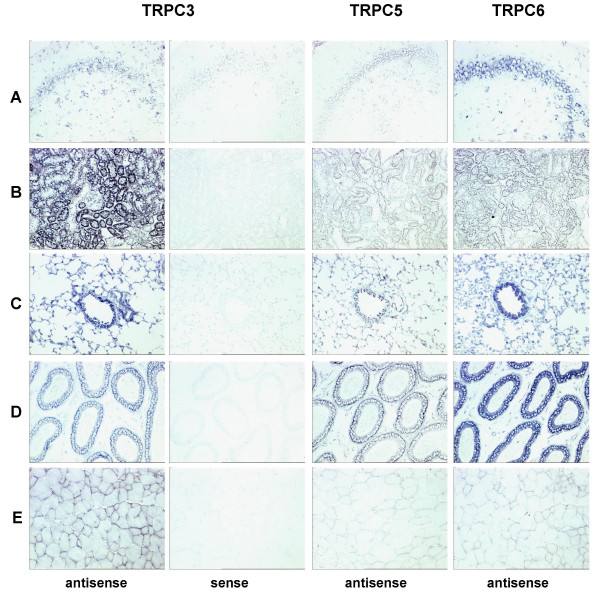
**In situ localization of three TRPC mRNAs in different mice organs using in situ hybridization**. *In situ *hybridization of TRPC3, TRPC5 and TRPC6 mRNA was performed with tissue sections of adult (100d) C57Bl/10SC mice. The two left hand columns of sections were incubated with the antisense probe for TRPC3 and the sense probe (control), respectively. For TRPC5 and TRPC6 only results with antisense probes are shown. Tissue sections: *A*: hippocampus, *B*: Kidney, *C*: Lung, *D*: epididymis, *E*: skeletal muscle. In all cases a methyl green counter-staining was performed. Magnification: × 200.

The *in situ *hybridization technique was used to localize mRNAs of TRPC3, 5 and 6 in certain cell types of the CNS and peripheral organs. Furthermore, the technique was used to visualize mRNA levels in these tissues. The experiments were performed with tissue slices of C57Bl/10 mice. For TRPC3, C5 and TRPC6 positive reactions were observed in the cytoplasm of neuronal cells in the C2 area of the hippocampus without any strong variation of expression levels (Fig. 4A). Sense probes applied as controls did not show positive results (Fig. 4, second column from left). In kidney, epithelial cells of the distal tubule showed intense staining of TRPC3 mRNA, while glomeruli and proximal tubules were negative. Distal tubules were also positive for TRPC5 and 6 but the staining intensity was much weaker (Fig. 4B). In lung tissue an intense staining was observed in bronchial epithelium for TRPC3 and C6 while TRPC5 detection was only slightly positive in these cells. For all three transcripts, pneumocytes showed a lesser reaction than bronchial epithelium, and even poorer staining was observed for blood vessels (Fig. 4C). Epididymis revealed strong expression of TRPC3 in the epithelium of the Ductuli epididymis and Ductuli efferentes testes while the other cell types were negative. The same result was obtained for TRPC5 and 6 in these cells (Fig. 4D). Cross sections of skeletal muscle showed most intense sarcolemmal staining for TRPC3 and less for TRPC6. The mRNA of TRPC5 was not detectable in these tissue slices (Fig. 4E). The results of *in situ *hybridization as well as staining intensities for many tissues and cell types are summarized in Table 4.

### Differences in TRP channel expression between mouse strains

**Figure 5:**
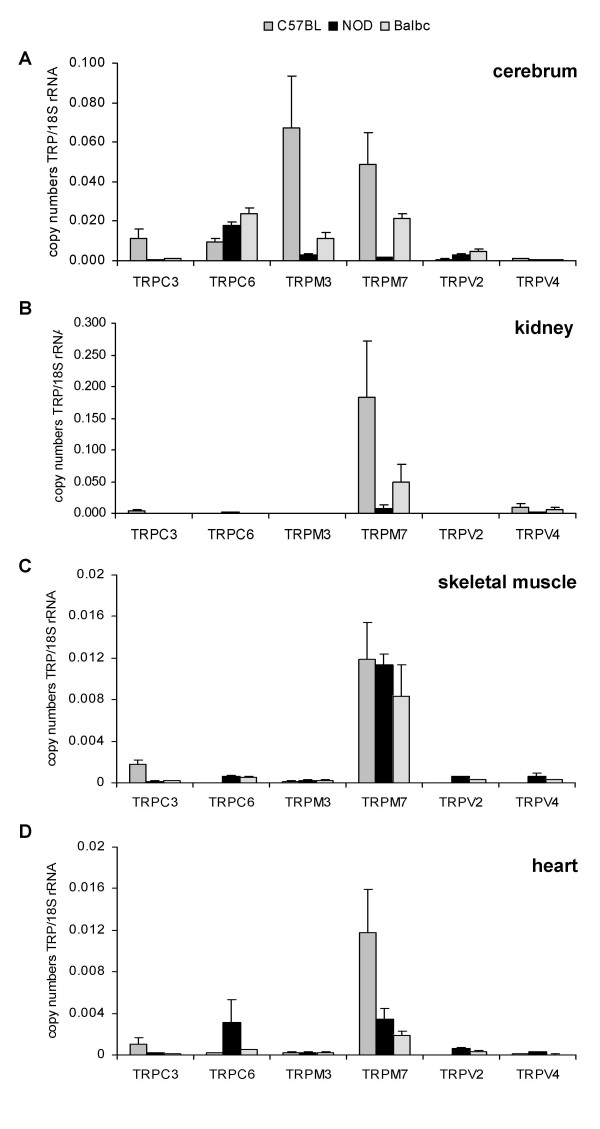
**Comparison of TRP channel mRNA levels in different mouse strains**. Expression of TRPC3, C6, M3, M7, V2 and V4 channels was determined by Real-time RT-PCR in selected tissues of three different mouse strains. Tissue samples were collected from adult (100 d) C57Bl/10SC (grey columns), NOD (black columns) and Balb/c mice (dotted columns). The mRNA levels of TRP channels are given in relation to 18S rRNA for cerebrum (A), kidney (B) skeletal muscle (C) and heart (D). Means ± S.E.M. are given in all cases for n = 3–10 samples.

In order to address the question of whether the findings on TRP channel gene expression can be generalized for the mouse, in addition to C57Bl/10 mice tissue we analyzed samples from animals of two other mouse strains, NOD and Balb/c. For most of the TRP channel transcripts and tissues similar levels were obtained for NOD and Balb/c mice compared to the C57Bl/10 strain (data not shown). However, some differences between the mouse strains became obvious. To ensure that possible polymorphisms in the primer or probe binding sites would not influence the amplification of the PCR products, we determined the amplification efficiencies for all transcripts and mouse strains shown in Fig. 5. For individual genes, PCR efficiencies varied only by several percent between the strains, indicating that they cannot account for the strain specific differences in gene expression. On this basis, TRPC3 expression was higher in some tissues, e.g. in cerebrum, skeletal muscle and heart of C57Bl/10 mice while TRPC6 expression was lower in the same tissues (Fig. 5). Data of NOD and Balb/c mice showed a high degree of congruence. In contrast to C57Bl/10, NOD and Balb/c mice showed a significant expression of TRPV2 and V4 in the heart and skeletal muscle. The very high expression of TRPM3 and M7 in cerebrum of C57Bl/10 mice was not observed in NOD and Balb/c. Furthermore, TRPM7 expression was very low in tissues of NOD mice compared to the C57Bl/10 and Balb/c strain (Fig. 5).

## Discussion

The TRP superfamily of ion channels is a structurally defined group of cation channels showing an enormous functional diversity [1]. Although several studies have shown the distribution and tissue specific expression of these channels in mammals, the relative importance of individual channels for certain tissues or cell types is widely unknown in most cases. The present study systematically quantifies the expression of all members of the TRPC, TRPV, TRPM and TRPA subfamilies in a broad spectrum of tissues.

Our results suggest a high relevance of TRPM channels in many murine tissues, since members of this subfamily showed the highest expression levels. These findings may qualify some studies that focused on members of the TRPC subfamily and tried to correlate gene expression with physiological function. Among the TRPMs, the chanzyme TRPM7 dominated over all other tested TRP members and seems to be ubiquitously expressed. As TRPM7 has a unique function for cellular Mg^2+ ^homeostasis [15,16] its wide distribution in all tested tissues looks plausible. In most brain areas the copy number of TRPM3 mRNA exceeded even that of TRPM7. In contrast to humans where TRPM3 shows a high expression in kidney [17], mouse kidney did not reveal extensive expression of TRPM3. This is in agreement with the results of Grimm and coworkers [18], who studied the tissue distribution of TRPM3 using northern blot analysis and immunochemical methods. Obviously, the osmotically regulated channel TRPM3 has different physiological relevance in different species.

Members of the TRPC subfamily were also widely distributed in murine tissues, but their overall expression was about one order of magnitude lower than that of the TRPMs. The dominant and most widely distributed isoform is TRPC3, which occurred in high concentrations in all tested brain areas. In addition, TRPC3 expression was obviously present in many other tissues such as liver, lung, kidney, ovary and testis. This is largely in agreement with results from rat tissues [11], only the absence of TRPC3 from rat liver showed a marked difference to our results. Further, we did not observe the strong expression of TRPC1 shown by Garcia and Schilling [11]. In many parts of human brain TRPC3 showed an at least tenfold higher expression than in peripheral tissues [12], a finding that is supported by this study in the mouse. Riccio and coworkers [12] also showed a high prevalence of TRPC1, C4, C5, and C7 in the CNS, i.e. a much stronger expression in all brain regions than in peripheral tissues. Only TRPC6 expression was in the same range in CNS as in peripheral tissues. Our study comes to similar results for the mouse. Furthermore, as levels of TRP channel mRNAs are normalized to that of 18S rRNA (Fig. 3), it is possible to estimate the relative importance of an individual TRP member for a certain tissue or brain region. For the cellular localization of TRP mRNAs in the CNS we used the *in situ *hybridization technique. In agreement with two previous publications [19,20] we detected TRPC3 mRNA and TRPC6 mRNA preferentially in Purkinje cells, neurons of basal ganglia and ventricle ependym, while TRPC5 mRNA was only found in a very small amount in any of these cell types. The morphometric results of the mRNA levels for TRPC3, C5 and TRPC6 displayed a good correlation between Real-time RT-PCR and *in situ *hybridization experiments.

Members of the TRPV subfamily showed an overall lower expression compared to members of TRPC and TRPM subfamilies. This is not surprising, since several of these channels serve as molecular sensors for pain and heat sensation and occur preferentially in sensory nerve terminals [1,6]. However, some characteristic exceptions have to be mentioned. High expressions of TRPV4 and TRPV5 were observed in kidney (Fig. 3). This finding is in agreement with earlier studies showing the importance of TRPV5 for calcium re-absorption in the kidney [21].

TRPA1 expression was even lower than that of TRPVs and more or less restricted to nervous tissue, ovary, testis and spleen. Low expression of TRPA1 in brain is compatible with its presumed function as a neuronal sensor molecule. Its occurrence in reproductive organs is described for the first time in this study.

Tissues or organs that mainly consist of muscle cells such as the aorta, skeletal muscle and heart showed an overall low expression of TRP channels. However, calcium conducting TRP channels may also be of great importance for muscle function, since contraction is controlled by calcium ions. The modulation of muscular calcium signaling by increased calcium entry through the sarcolemma can have consequences for muscle function and may cause dysfunction and disease [9]. For members of the TRPC subfamily the expression in smooth muscle and tissue preparations such as the aorta have been well investigated. Signals of TRPC1, C3, C4, C5 and C6 have been reported in mouse and rat aorta, while TRPC2 and C7 transcripts were not observed [7]. Our study confirms many of these results. In addition, we observed the presence of TRPC2 in the aorta, but very low signals for TRPC4 and C5. Furthermore, other members of the TRP family may be of relevance for aortic smooth muscle. We found TRPV4 expression almost as high as that of the most abundant TRPC member, TRPC3. TRPC3 and TRPV4 expression was even exceeded by TRPM5 and TRPM7 (Fig. 3).

In human heart muscle several TRPC transcripts have been detected. TRPC1, C4, C5, and C6 mRNAs show moderate levels compared to CNS [12]. This study comes to the result that among TRPC family members only TRPC1, C3 and C6 occur in mouse heart at significant levels. We found only traces of TRPC4 and C5 mRNAs. Differences between mouse and human heart or the fact that TRPC4 and C5 are not constitutively expressed may be responsible for these findings. Recently, the upregulation of TRPC4 and C5 was shown in cardiomyocytes in response to inhibition of SERCA expression. In this model TRPC4 and C5 seem to be induced after insufficient function of SR calcium accumulation [22]. Using *in situ *hybridization experiments we were able to localize the mRNAs for TRPC3 and TRPC6 in cardiac muscle cells but not in the endothelium of cardiac blood vessels. It is known that the subgroup of TRPC3/C6/C7 can assemble into homo- and heterotetramers [23]. As TRPC3 and C6 are expressed at significant levels in mouse heart, these two channels have the potential to form several Ca^2+ ^conducting non selective cation channels in myocytes and contribute to cardiac Ca^2+ ^homeostasis.

Surprisingly, cardiac expression levels of TRP mRNAs showed considerable variability among different mouse strains. In C57Bl mice TRPC3 was dominantly expressed in heart muscle whereas TRPC6 was the major isoform in NOD and Balb/c hearts. Heterogeneity was also observed for TRPV2 expression. This mRNA was only found in NOD and Balb/c hearts and almost absent in the C57Bl strain. Recently, the presence of TRPV2 in the sarcolemma of cardiac muscle has been reported. Further, the cardiac-specific overexpression of TRPV2 caused a cardiomyopathy due to cellular Ca^2+ ^overload in a transgenic mouse model. The severity of the cardiomyopathy was roughly related to sarcolemmal TRPV2 levels [24]. These data show that TRPV2 can be an important element of cardiac Ca^2+ ^homeostasis. Besides TRPCs and TRPV2, other members of the TRP family were found to be expressed in heart muscle, e.g. TRPV4, V6, M4 and M7. This is in accordance with previous studies showing the expression of TRPV4 [25], TRPV6 [26]and TRPM4 [4] in murine heart.

In addition to earlier studies that focussed on the TRPC subfamily [27,28], we investigated expression levels of TRPV and TRPM channels in skeletal muscle. Two reports described the presence of TRPC1, C2, C3, C4 and C6 mRNAs by standard RT-PCR and showed that the corresponding channel proteins are localized in the sarcolemma [27,28]. These data are in agreement with our results, however we suggest a dominant role of TRPC3 in mouse muscle, since the TRPC3 transcript occurs in much higher concentrations than those of the other TRPC members. In addition, mRNAs of TRPV3, V4 and V6 as well as TRPM3, M4 and M7 were found in considerable concentrations in several mouse skeletal muscles including diaphragm.

Apart from RT-PCR studies, databases containing expressed sequence tags (ESTs) can be used to estimate gene expression. The relation of the ESTs of a certain gene to the total number of ESTs derived from a tissue provides a measure for gene expression [31]. However, TRP transcripts code for membrane proteins of low abundance and constitute only a small fraction of the cellular transcriptome. Therefore it is not surprising that ESTs derived from TRP RNAs occur at very low frequencies. EST databases are not very informative for tissues such as skeletal muscle and heart, since the number of hits for individual TRP transcripts is often 0 or 1. Large numbers of ESTs exist for certain brain regions. In combination with the fact of a rather high TRP channel expression in nervous tissue EST frequencies may reflect gene expression and can be compared to RT-PCR data. For cerebrum and cerebellum our RT-PCR data (Fig. 3) widely agree qualitatively with the occurrence of EST frequencies included in UniGene. Both sets of data show high expression most TRPC members, TRPM3, 4 and 7, followed by TRPV2, 4 and 6. However, current EST data show quantitative differences to the RT-PCR data and reveal some unexpected negative results. For example, in UniGene there is no hit for TRPC1 in cerebrum and cerebellum is negative for TRPV4 and 6. Thus, to date RT-PCR seems to be the superior method for studying gene expression of low abundance transcripts such as TRP channel mRNAs.

## Conclusion

We have characterized the tissue specific mRNA expression of 22 TRP ion channels of four major subfamilies. We conclude that transcripts coding for members of the TRPC, TRPV, TRPM and TRPA subfamilies are present in a broad spectrum of murine tissues. In addition to well studied tissues we detected many TRP isoforms in poorly investigated tissues such as skeletal muscle, heart and reproductive organs. Expression levels of members of the TRPM subfamily were generally higher than those of TRPC and TRPV subfamilies indicating important physiological roles of TRPMs. For some TRPC members the data obtained by quantitative RT-PCR were largely confirmed by in situ hybridization. In view of the mouse as the major model organism of man, differences between TRP channel expression in human and murine tissues are of major interest. This study offers a guideline for the investigation of the physiological relevance of individual members of almost the whole TRP channel superfamily.

## Methods

### Animals

Mice of the inbred strains C57Bl/10SC (originally obtained from Charles River, Sulzfeld, Germany), Balb/c and NOD (originally obtained from Taconic Europe Inc., Ry, Denmark) were used for the study. All animals were bred in the Department of Laboratory Animal Science of the Medical Faculty, University of Greifswald. Adult mice (100 d) of either sex were killed using ether inhalation in a manner approved by the institutional animal ethics committee. Tissue samples were removed quickly and frozen in liquid nitrogen.

### Cell lines

The mouse skeletal muscle cell line C2C12 was obtained from the Department of General Physiology of the University of Ulm. Cells were routinely grown in Dulbecco's modified Eagle's medium containing 10% fetal calf serum, 1% L-glutamine and 1% penicillin-streptomycin (all from Sigma) in a humidified 5% CO_2 _atmosphere at 37°C. Cells were grown in a low density of 5 × 10^5 ^cells/35-mm culture dishs and submitted to polymerase chain reaction (RT-PCR).

### RNA extraction and reverse transcription

Total RNA was isolated using guanidinium-isothiocyanate (RNeasy Mini Kit, Qiagen, Hilden, Germany) and RNA concentration was determined by UV absorbance measurements. An amount of 200 ng total RNA was reverse transcribed using random hexamer primers and the TaqMan Reverse Transcription Reagents (PE Applied Biosystems, Weiterstadt, Germany).

### Standard RT-PCR

PCR was performed with Taq Polymerase (Qiagen) for 40 cycles using a Thermal Cycler Px2 (Thermo Electron, Dreieich, Germany). Primers used for amplification of murine TRP and GAPDH gene fragments are shown in Table 1. For standard RT-PCR an amount of 8 ng reverse transcribed RNA was used in a 50 μl reaction volume. The thermocycler was programmed to apply an initial cycle consisting of 94°C denaturation for 5 min, followed by 40 cycles of 54°C annealing for 15 s, 72°C elongation for 30 s and 94°C denaturation for 15 s. A final elongation step at 72°C for 5 min was included. A "no-template control" with water was performed alongside all experiments. Parallel PCR analysis was run for the housekeeping gene GAPDH to normalize data for differences in mRNA quantity and integrity. All primers were obtained from TIBMOLBIOL (Berlin, Germany).

### Preparation of DNA fragment standards

For real-time quantification by TaqMan, DNA fragments containing the target sequences of TRP channels and those of 18S rRNA were cloned in the pGEM-T-Easy cloning vector (Promega, Germany) according to the manufacturer's instructions and sequenced. Sequence identities were established by searching the databases using the NCBI BLAST program.

### TaqMan RT-PCR

To quantify the expression of mouse TRP channel genes, we applied the TaqMan PCR system using the 5700 Sequence Detector (Applied Biosystems). Gene-specific TaqMan PCR primers and probes for murine TRP channels were purchased from PE Applied Biosystems (Table 2) or TIBMOLBIOL (Table 3), whereby each probe was synthesized with a fluorescent 5'-reporter dye (FAM: 6-carboxy-fluorescein) and a 3'-quencher dye (TAMRA: 6-carboxy-tetramethyl-rhodamine). Parallel TaqMan PCR assays for each gene target were performed with cDNA samples and genomic standards. Reaction mixtures contained 1× TaqMan Universal PCR Master Mix (Applied Biosystems), 1× murine specific Primer and Probe Mixture (see Tables 1, 2, 3) or 1× Eukaryotic 18S rRNA Endogenous control (Applied Biosystems). To quantify TRP channel expression an amount of 8 ng reverse transcribed RNA was used in a 20 μl reaction volume. To amplify 18S rRNA (internal control) only 20 pg were used. PCR products were amplified (50°C, 2 min; 95°C 10 min followed by 40 cycles of 95°C, 15 s and 60°C, 1 min) and analysed on a real-time PCR cycler (SDS 5700, Applied Biosystems). Absolute copy numbers of TRP channel transcripts and 18S cDNA were determined using calibration curves generated with cloned PCR fragment standards. Copy numbers of individual TRP channel transcripts are given in relation to those of 18S cDNA. A "no-template control" with water was performed parallel in all experiments. Each series of experiments was performed twice.

### Preparation of riboprobes for in situ hybridization

For TRPC3, TRPC5 and TRPC6 cDNA fragments of about 200 to 300 bp length (TRPC3 [GenBank: NM_019510] position nt +1856 to +2173; TRPC5 [GenBank: NM_009428] position nt +2472 to +2686, TRPC6 [GenBank: NM_013838] position nt +2181 to +2507) were cloned into the pGEM-T-Easy cloning vector (Promega, Germany). The fragments were labeled with DIG (digoxigenin) by *in vitro *transcription using the *DIG RNA labeling Kit (*SP6/T7, Roche Biochemicals, Germany). The antisense cRNA was used for the detection of the mRNA whereas the sense cRNA probe served as control.

### In situ hybridization analysis on paraffin sections

Non-radioactive *in situ *hybridization was performed with paraffin sections (4 μm) which had been fixed in 4 % paraformaldehyde. Sections were rehydrated and permeabilized by pepsin digestion (750 μg/ml pepsin in 0.2 M HCl, 37°C, 30 min). Post-fixation (paraformaldehyde 4 %, 20 min, 4°C) was followed by acetylation using 0.4 % acetic anhydride in triethanolamine (0.1 M, pH8.0, 15 min). After washing with 50 % formamide in 1.5 % SSPE (20 × SSPE: 3.6 M NaCl, 0.2 M NaH_2_PO_4_, 0.2 M EDTA, pH 7.4) the sections were prehybridized for 1 h at 56°C in a solution containing 50 % formamide and 50 % solution D (4 M guanidine thiocyanate, 25 mM sodium citrate, pH 7.0), 0.5% blocking reagent (Roche Biochemicals, Germany) and 210 μg/ml t-RNA. After hybridization for 12–16 h with prehybridization solution containing 125 ng digoxigenin (DIG)-labeled cRNA probe and washing with 2 × SSC (20 × SSC: 3 M NaCl, 0.3 M sodium citrate; pH 7.4), sections were incubated with blocking reagent (Roche Biochemicals, Germany). Bound riboprobe was visualized by incubation with alkaline phosphatase-conjugated anti-DIG antibody (Roche Biochemicals, Germany) and subsequent substrate reaction containing 5-bromo-4-chloro-3-indolyl phosphate/nitroblue-tetrazolium chloride. The level of mRNA expression was determined in a blinded fashion in a single run with identical staff, equipment, and chemicals. Analysis of the stained sections was carried out by two independent investigators and staining intensity was scored ± (very weak reaction), + (weak reaction), ++ (moderate reaction), and +++ (strong reaction).

## Authors' contributions

CKK contributed to the design and coordination of the study assembled figures and worked on the manuscript. CKK further performed TaqMan RT-PCR experiments. FB carried out in-situ hybridization experiments. JK carried out tissue preparation, RNA isolation and standard RT-PCR. HB contributed to the design and planning of the study, to the discussion and interpretation of results and to the writing of the manuscript.

## Figures and Tables

**Table 1:** Primers used for standard RT-PCR

**Gene**	**Accession number, product size & references**	**Sequence 5'-3'**
TRPC1	NM_011643 371 bp [11]	**for: **CAAGAT TTTGGGAAATTTCTGG **rev: **TTTATCCTCATGATTTGCTAT

TRPC2	NM_011644 326 bp [29]	**for: **GATCCGGTTCATGTTCATCCT **rev: **GAGCGAGCAAACTTCCACTC

TRPC3	NM_019510 317 bp [11]	**for: **TGACTTCCGTTGTGCTCAAATATG **rev: **CCTTCTGAAGCCTTCTCCTTCTGC

TRPC4	NM_016984 414 bp [11]	**for: **TCTGCAGATATCTCTGGGAAGGATGC **rev: **AAGCTTTGTTCGAGCAAATTTCCATTC

TRPC5	NM_009428 339 bp [11]	**for: **ATCTACTGCCTAGTACTACTGGCT **rev: **CAGCATGATCGGCAATGAGCTG

TRPC6	NM_013838 326 bp [11]	**for: **AAAGATATCTTCAAATTCATGGTC **rev: **CACGTCCGCATCATCCTCAATTTC

TRPC7	NM_012035 692 bp	**for: **CGTGCTGTATGGGGTTTATAATG **rev: **GCTTTGGAATGCTGTTAGAC

TRPV1	NM_001001445 320 bp	**for: **GCATCTTCTACTTCAACTTCTTCGTC **rev: **CCACATACTCCTTGCGATGGC

TRPV2	NM_011706 427 bp	**for: **CGGCACTTCCTCTCT **rev: **GTCGGTCACGGTCAA

TRPV3	NM_145099 298 bp	**for: **CAAGGACTGCCACCACCATC **rev: **CATCACAGTTGCCAGAGAGG

TRPV4	NM_022017 249 bp	**for: **GAGTCCTCAGTAGTGCCTGG **rev: **CAACAAGAAGGAGAGCAGTC

TRPV5	NM_001007572 172 bp [30]	**for: **CGTTGGTTCTTACGGGTTGAA **rev: **GTTTGGAGAACCACAGAGCCTCTA

TRPV6	NM_022413 318 bp	**for: **AACCAGCCTTCCACC **rev: **CCTCCATTAGCACCA

TRPM1	NM_018752 446 bp	**for: **TATCCTATGACACCAAGCCA **rev: **GGTCTTCCTTATTCTCCACG

TRPM2	NM_138301 215 bp	**for: **CAGATCCCAACCTACATTGACG **rev: **GAAGGTGTAGTTGAACATGGCGA

TRPM3	NM_177341 497 bp	**for: **CCTGTTCTTCTGGCA **rev: **GCTTCTCTGGCTCCT

TRPM4	NM_175130 176 bp	**for: **CCCTGAGGATGGTGTTGAGT **rev: **AGGAGCACTGGGATGTCAAT

TRPM5	NM_020277 360 bp	**for: **CAGATACTGAGGATGGCTGG **rev: **GGATCTTGGTGGATGTGCTA

TRPM6	NM_153417 219 bp	**for: **CCAGGTGCCGGTAATAACA **rev: **CTCTTGTGGCTGCCTTAGGT

TRPM7	NM_021450 295 bp	**for: **CGGAGCTGGTCGCACAATTA **rev: **CCTGGAAGACATCTGTGAGG

TRPM8	NM_134252 344 bp	**for: **TCCACGAGACTTCTTCACTT **rev: **GTGTATCTGAGGTCAGTCTT

GAPDH	NM_008084 451 bp	**for: **ACCACAGTCCATGCCATCAC **rev: **TCCACCACCCTGTTGCTGTA

Primers described in the literature are indicated (references). All other primers were newly designed.

**Table 2:** Information about real-time RT-PCR primers and probes for indicated ion channel transcripts

**Gene**	**Accession number**	**Assay-on-Demand**
TRPC1	NM_011643	Mm00441975_m1
TRPC2	NM_011644	Mm00441984_m1
TRPC5	NM_009428	Mm00437183_m1
TRPC6	NM_013838	Mm00443441_m1
TRPC7	NM_012035	Mm00442606_m1
TRPV2	NM_011706	Mm00449223_m1
TRPV3	NM_145099	Mm00454996_m1
TRPV4	NM_022017	Mm00499025_m1
TRPV6	NM_022413	Mm00499069_m1
TRPM1	NM_018752	Mm00450619_m1
TRPM5	NM_020277	Mm00498453_m1
TRPM7	NM_021450	Mm00457998_m1
TRPM8	NM_134252	Mm00454566_m1
TRPA1	NM_177781	Mm00625268_m1

The sets of probes and primers for detection of TRP channel mRNAs by TaqMan RT-PCR were obtained from PE Applied Biosystems.

**Table 3:** Sets of real-time RT-PCR primers and probes for detection of TRP ion channel transcripts

**Gene**	**Accession number**	**Sequence 5'-3'**
TRPC3	NM_019510	**for: **CCAAGCTGGCCAACATAGAG **rev: **GGCAAGTTTGACACGACTCA **probe: **actcggaggaggtggaagccattc

TRPC4	NM_016984	**for: **TGGAGTGGATGATATTACCG **rev: **CCACATGTCCCATGATTC **probe: **TGTGATGAACTCCTTGTATCTGGCAACAA

TRPV1	NM_001001445	**for: **GCATCTTCTACTTCAACTTCTTCGTC **rev: **CCACATACTCCTTGCGATGGC **probe: **CAAACTCTTGAGGGATGGTCGCCTCT

TRPV5	NM_001007572	**for: **CAAGAAGAAAGAGGCTCGAC **rev: **AATGACTGTCACCAACTCCC **probe: **CCGTACACATGTTCGAGATAACACCATCA

TRPM2	NM_138301	**for: **CAGATCCCAACCTACATTGACG **rev: **GAAGGTGTAGTTGAACATGGCGA **probe: **ACCAGTGCAGCCCCAATGGCA

TRPM3	NM_177341	**for: **CAAAGATGACATGCCCTATATGA **rev: **CTTTCTTTCTGGATGATTCCC **probe: **ATGAAGAGGACATGGAGCTAACAGCAA

TRPM4	NM_175130	**for: **CCCTGAGGATGGTGTTGAGT **rev: **AGGAGCACTGGGATGTCAAT **probe: **CTTCTTGGTGGATGATGGCACC

TRPM6	NM_153417	**for: **CCAGGTGCCGGTAATAACA **rev: **CTCTTGTGGCTGCCTTAGGT **probe: **CAGTTGAATGCAGAGCCAGGAGAAAC

The sets of PCR primers and TaqMan fluorogenic probes were obtained from TIBMOLBIOL.

**Table 4:** In situ localization of TRPC3, TRPC5 and TRPC6 transcripts in different mouse organs

**tissue**	**TRPC3**	**TRPC5**	**TRPC6**
Skeletal muscle			
• sarcolemma	+	neg.	±
• sarcoplasmic reticulum	neg.	neg.	neg.
• myofibrils	neg.	neg.	neg.

Heart			
• cardiac muscle cells	+	neg.	±
• blood vessel endothelium	neg.	neg.	neg.

Aorta			
• Tunica muscularis	+	+	n. d.
• endothelium	+	neg.	n. d.

small intestine			
• smooth muscle layer	neg.	±	neg.
• submucosa	+	±	±
• enterocytes of the villi	++	+	++
• enterocytes of the crypts	+++	+++	++

Kidney			
• glomeruli	±	neg.	neg.
• distal tubule	+++	±	++
• proximal tubule	neg.	neg.	neg.

Lung			
• bronchial epithel	+++	+	++
• pneumocytes	+	±	+
• blood vessel endothelium	+	neg.	+

Liver			
• hepatocytes	+++	++	++
• epithel of the Ductus interlobularis bilifer	+++	++	++

Spleen			
• red pulp	++	neg.	neg.
• white pulp	neg.	++	++

Pancreas			
• exocrine acini	+++	+++	+++
• islet of Langerhan's	neg.	neg.	neg.
• duct system	++	neg.	neg.
• blood vessel endothelium	neg.	neg.	neg.

testis			
• leydig cells	+	+	++
• sertoli cells	neg.	neg.	neg.
• spermatogonia/spermatocytes	neg.	neg.	+
• spermatozoa	neg.	neg.	++

Epididymis			
• epithelium of the Ductuli efferentes testis	+++	++	++
• epithelium of the Ductus epididymidis	+	++	++
• spermatozoa	neg.	neg.	neg.

Uterus			
• endometrium – glandular epithelial cells	++	+++	+++
• myometrium	neg.	++	+

fallopian tube			
• smooth muscle layer	neg.	±	neg.
• tubal epithelium	+	+	+

Ovary			
• cortex			
- granulosa cells	++	+	++
- theca cells	neg.	±	+
- corpus luteum	neg.	±	+
• medulla	neg.	neg.	+

CNS			
• spinal ganglia	+++	+	n. d.
• cortex	++	±	+
• hippocampus	+	neg.	+
• ventricle ependym	+++	±	++
• basal ganglia	++	neg.	++
• cerebellum			
- Purkinje cells	++	±	+++
- Stratum granulosum	+	neg.	+

The *in situ *hybridization technique was applied to tissue sections as shown in Fig. 3. The obtained signals were classified into six categories: negative (neg.), very weak (±), weak (+), moderate (++), strong (+++) or not determined (n. d.).

## References

[B1] Montell C (2005). The TRP superfamily of cation channels. Sci STKE.

[B2] Montell C, Birnbaumer L, Flockerzi V, Bindels RJ, Bruford EA, Caterina MJ, Clapham DE, Harteneck C, Heller S, Julius D, Kojima I, Mori Y, Penner R, Prawitt D, Scharenberg AM, Schultz G, Shimizu N, Zhu MX (2002). A unified nomenclature for the superfamily of TRP cation channels. Mol Cell.

[B3] Lu G, Henderson D, Liu L, Reinhart PH, Simon SA (2005). TRPV1b, a functional human vanilloid receptor splice variant. Mol Pharmacol.

[B4] Murakami M, Xu F, Miyoshi I, Sato E, Ono K, Iijima T (2003). Identification and characterization of the murine TRPM4 channel. Biochem Biophys Res Commun.

[B5] Harteneck C (2003). Proteins modulating TRP channel function. Cell Calcium.

[B6] Clapham DE (2003). TRP channels as cellular sensors. Nature.

[B7] Beech DJ, Muraki K, Flemming R (2004). Non-selective cationic channels of smooth muscle and the mammalian homologues of Drosophila TRP. J Physiol.

[B8] Clapham DE, Runnels LW, Strubing C (2001). The TRP ion channel family. Nat Rev Neurosci.

[B9] Berchtold MW, Brinkmeier H, Müntener M (2000). Calcium ion in skeletal muscle: its crucial role for muscle function, plasticity, and disease. Physiol Rev.

[B10] Berridge MJ, Bootman MD, Roderick HL (2003). Calcium signalling: dynamics, homeostasis and remodelling. Nat Rev Mol Cell Biol.

[B11] Garcia RL, Schilling WP (1997). Differential expression of mammalian TRP homologues across tissues and cell lines. Biochem Biophys Res Commun.

[B12] Riccio A, Medhurst AD, Mattei C, Kelsell RE, Calver AR, Randall AD, Benham CD, Pangalos MN (2002). mRNA distribution analysis of human TRPC family in CNS and peripheral tissues. Brain Res Mol Brain Res.

[B13] Yip H, Chan WY, Leung PC, Kwan HY, Liu C, Huang Y, Michel V, Yew DT, Yao X (2004). Expression of TRPC homologs in endothelial cells and smooth muscle layers of human arteries. Histochem Cell Biol.

[B14] Kitahara T, Li HS, Balaban CD (2005). Changes in transient receptor potential cation channel superfamily V (TRPV) mRNA expression in the mouse inner ear ganglia after kanamycin challenge. Hear Res.

[B15] Wolf FI (2004). TRPM7: channeling the future of cellular magnesium homeostasis?. Sci STKE.

[B16] Fleig A, Penner R (2004). The TRPM ion channel subfamily: molecular, biophysical and functional features. Trends Pharmacol Sci.

[B17] Lee N, Chen J, Sun L, Wu S, Gray KR, Rich A, Huang M, Lin JH, Feder JN, Janovitz EB, Levesque PC, Blanar MA (2003). Expression and characterization of human transient receptor potential melastatin 3 (hTRPM3). J Biol Chem.

[B18] Grimm C, Kraft R, Sauerbruch S, Schultz G, Harteneck C (2003). Molecular and functional characterization of the melastatin-related cation channel TRPM3. J Biol Chem.

[B19] Mori Y, Takada N, Okada T, Wakamori M, Imoto K, Wanifuchi H, Oka H, Oba A, Ikenaka K, Kurosaki T (1998). Differential distribution of TRP Ca2+ channel isoforms in mouse brain. Neuroreport.

[B20] Mizuno N, Kitayama S, Saishin Y, Shimada S, Morita K, Mitsuhata C, Kurihara H, Dohi T (1999). Brain Res Mol Brain Res.

[B21] van Abel M, Hoenderop JG, Bindels RJ (2005). Molecular cloning and characterization of rat trp homologues from brain. Naunyn Schmiedebergs Arch Pharmacol.

[B22] Seth M, Sumbilla C, Mullen SP, Lewis D, Klein MG, Hussain A, Soboloff J, Gill DL, Inesi G (2004). Sarco(endo)plasmic reticulum Ca2+ ATPase (SERCA) gene silencing and remodeling of the Ca2+ signaling mechanism in cardiac myocytes. Proc Natl Acad Sci USA.

[B23] Dietrich A, Mederos YSM, Kalwa H, Storch U, Gudermann T (2005). Functional characterization and physiological relevance of the TRPC3/6/7 subfamily of cation channels. Naunyn Schmiedebergs Arch Pharmacol.

[B24] Iwata Y, Katanosaka Y, Arai Y, Komamura K, Miyatake K, Shigekawa M (2003). A novel mechanism of myocyte degeneration involving the Ca2+-permeable growth factor-regulated channel. J Cell Biol.

[B25] Strotmann R, Harteneck C, Nunnenmacher K, Schultz G, Plant TD (2000). OTRPC4, a nonselective cation channel that confers sensitivity to extracellular osmolarity. Nat Cell Biol.

[B26] Nijenhuis T, Hoenderop JG, van der Kemp AW, Bindels RJ (2003). Localization and regulation of the epithelial Ca2+ channel TRPV6 in the kidney. J Am Soc Nephrol.

[B27] Weigl L, Zidar A, Gscheidlinger R, Karel A, Hohenegger M (2003). Store operated Ca2+ influx by selective depletion of ryanodine sensitive Ca2+ pools in primary human skeletal muscle cells. Naunyn Schmiedebergs Arch Pharmacol.

[B28] Vandebrouck C, Martin D, Colson-Van Schoor M, Debaix H, Gailly P (2002). Involvement of TRPC in the abnormal calcium influx observed in dystrophic (mdx) mouse skeletal muscle fibers. J Cell Biol.

[B29] Gailly P, Colson-Van Schoor M (2001). Involvement of trp-2 protein in store-operated influx of calcium in fibroblasts. Cell Calcium.

[B30] Van Cromphaut SJ, Dewerchin M, Hoenderop JG, Stockmans I, Van Herck E, Kato S, Bindels RJ, Collen D, Carmeliet P, Bouillon R, Carmeliet G (2001). Duodenal calcium absorption in vitamin D receptor-knockout mice: functional and molecular aspects. Proc Natl Acad Sci USA.

[B31] Unigene database.

